# Video-call based newborn triage system for local birth centres can be established without major instalment costs using commercially available smartphones

**DOI:** 10.1038/s41598-020-64223-w

**Published:** 2020-05-05

**Authors:** Junichiro Okada, Tadashi Hisano, Mitsuaki Unno, Yukari Tanaka, Mamoru Saikusa, Masahiro Kinoshita, Eimei Harada, Sachiko Iwata, Osuke Iwata

**Affiliations:** 1 Division of Neonatology, St. Mary’s Hospital, Fukuoka, Japan; 2 Center for Human Development and Family Science, Department of Neonatology and Pediatrics, Nagoya City University Graduate School of Medical Sciences, Nagoya, Aichi Japan; 3 Centre for Developmental and Cognitive Neuroscience, Department of Paediatrics and Child Health, Kurume University School of Medicine, Fukuoka, Japan

**Keywords:** Paediatric research, Neonatology

## Abstract

Neonates often develop transition problems after low-risk birth, precise assessment of which is difficult at primary birth centres. The aim of this study was to assess whether a video triage system can be established without a specially designed communication system between local birth centres and a tertiary neonatal intensive care unit in a region with a population of 700,000. 761 neonates who were referred to a tertiary neonatal intensive care unit were examined. During period 1 (April 2011-August 2015), only a voice call was available for consultations, whereas, during period 2 (September 2015-December 2017), a video call was additionally available. The respiratory condition was assessed based on an established visual assessment tool. A video consultation system was established by connecting personal smartphones at local birth centres with a host computer at a tertiary neonatal intensive care centre. During period 2, video-based triage was performed for 42.4% of 236 consultations at 30 birth centres. Sensitivity and specificity for predicting newborns with critical respiratory dysfunction changed from 0.758 to 0.898 and 0.684 to 0.661, respectively. A video consultation system for ill neonates was established without major instalment costs. Our strategy might improve the transportation system in both high- and low-resource settings.

## Introduction

Approximately 10% of newborns require some form of special care before spontaneous breathing is established^1^. To improve the outcomes of high-risk foetuses, antenatal diagnoses and maternal transportation to higher-level birth centres have been encouraged^2^. However, for newborns born near term and at term, who require advanced care because of transition failure, abnormal clinical signs are often unnoticed until much later after birth^3^. Dissemination of an evidence-based program of neonatal cardiopulmonary resuscitation (NCPR) led to improved support for ill newborns worldwide^4,5^. However, newborns who cannot establish spontaneous breathing need to be transferred to neonatal intensive care units (NICUs) for advanced care support. Indeed, a large-scale survey in Japan conducted in 2012 showed that 20.5% of 55,331 newborns admitted to NICUs were transferred from primary/secondary birth centres after birth^6^. Although careful assessments are essential for precise triage decisions, currently, there is no established system for evaluating the conditions of newborns before transportation, potentially leading to diagnostic discordance between the reason for transport and final diagnosis, as reported in paediatric transport^7^. Our group has previously developed a visual assessment scale that can objectively predict which ill newborns have low respiratory compliance^8^. Implementing such a scale with a video call–based consultation system might improve the safety of newborns who would require additional support during transportation.

For adults, telemedicine using a video call has been proposed for better control of chronic diseases^9,10^, expert diagnosis of radiographic images^11,12^ and consultations for advanced treatments^13–15^. Even in the perinatal field, live images, such as fundoscopy and cardiac ultrasound, are shared between remote institutions to involve local physicians and experts in the field^16–19^. Fang and colleagues successfully developed a telemedicine-based consultation system for neonatal resuscitation to improve patient access to expertise in neonatology and reduce unnecessary transfers of newborns to a higher level of care^20^. While these reports are encouraging, other studies suggested that further improvements are required for the security and reliability of the application and network used^21,22^. In addition, few of these systems survived beyond the test phase, presumably because of the maintenance cost and incompatibility with other communication tools/networks^18,23^. Given that video calls can now be easily performed using mobile phones, a flexible video call-based consultation network might be established without major installation costs.

The aim of this study was to assess whether a consultation system for ill newborns, which incorporated video calls, can be established in primary/secondary birth centres and a tertiary NICU in a region with a population of 700,000 and to determine if this system can increase true-positive triage of clinically unstable newborns without increasing the use of medical resources required for the transportation.

## Results

Values are shown as mean (SD) unless otherwise specified. The distance of one-way transportation was 17.4 (16.9) km. Twenty-seven (90%) units used the video call within the first 12 months of period 2. Background variables of the 761 newborns were; gestational age, 38.5 (2.1) weeks; birth weight, 2,800 (536) g; and postnatal age at referral, 16.5 (18.1) h. One hundred seventy-five newborns (23.0%) were born via induced delivery (both vaginal and caesarean). Three hundred eighty-seven newborns (50.9%) were referred during the weekday daytime. Primary clinical problems at referral were respiratory problems (36.9%), gastrointestinal problems (13.5%), preterm birth and/or low birth weight (13.5%), cardiac problems (8.3%), congenital anomalies (5.0%), jaundice (4.7%), hypothermia and signs of being unwell (4.6%), fever and/or infection (4.1%), birth asphyxia (3.4%), hypoglycaemia (3.2%), neurological problems (2.5%) and other disorders (0.3%; including 1 trauma/haematoma and 1 haematologic disease).

At the time of referral, 319 newborns (41.9%) were estimated to be clinically unstable based on the voice/video call. Subsequently, IPPV and NIPPV were required for 19.7% (28.5% and 14.6%, respectively, for newborns, whose primary problems at referral were respiratory and non-respiratory symptoms) and 5.9% (6.0% and 5.8%, respectively, for newborns, whose primary problems at referral were respiratory and non-respiratory symptoms) of newborns, respectively, during or <6 h after transportation.

**Figure 1:**
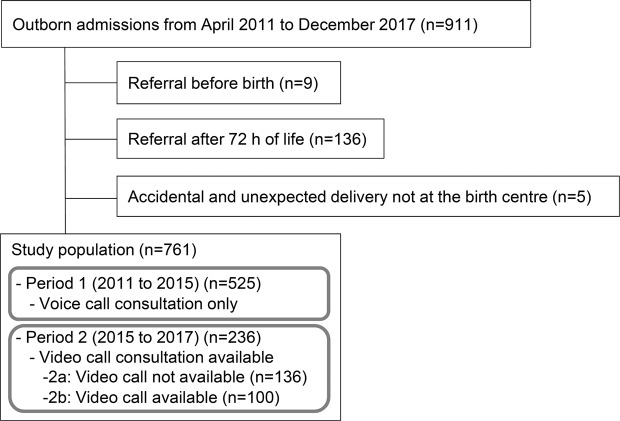
Profile of the study population.

All newborns survived and were discharged home. Of 761 newborns, 525 were referred during period 1 (Fig. 1). During period 2, 236 newborns were referred, and video calls were used for 100 of them (42.4%) (Table 1). Ninety-four video calls used FaceTime (Apple Inc., Cupertino, CA, USA), whereas six video calls were made by Skype (n = 3; Microsoft Corporation, Redmond, WA, USA), Zoom (n = 2; Zoom Video Communications, San Jose, CA, USA) or Google Hangouts (n = 1; Google LLC, Mountain View, CA, USA); none used Line Phone (Line Corporation, Tokyo, Japan) and Facebook Messenger (Facebook Inc., Menlo Park, CA, USA). Six obstetricians did not own a smartphone, but we were able to make video calls to the smartphones of the nursing/midwifery staff members. Video calls were not used for the remaining 136 newborns because of the following reasons: stable conditions appeared evident from the information provided during the voice call (n = 92); critical conditions appeared evident from the information provided during the voice call (n = 37); staff members in charge were unfamiliar with the video call system (n = 7). Background clinical variables did not differ between periods 1 and 2 or between periods 2a and 2b (Table 1).

**Table 1:** Clinical backgrounds of newborns registered during each study period with and without video calls.

	Period 1	Period 2		
		Whole cohort	a Video call (−)	b Video call (+)
	n = 525	n = 236	n = 136	n = 100
Gestational age (week)	38.5 ± 2.1	38.5 ± 2.2	38.2 ± 2.6	38.9 ± 1.5
Body weight at birth (g)	2806 ± 526	2788 ± 557	2703 ± 612	2904 ± 450
Male sex	307 (58.5%)	136 (63.6%)	80 (58.8%)	56 (56.0%)
All caesarean section	128 (24.4%)	72 (30.5%)	42 (30.9%)	30 (30.0%)
Emergency caesarean section	64 (12.2%)	29 (12.3%)	19 (14.0%)	10 (10.0%)
Elective caesarean section	64 (12.2%)	43 (18.2%)	23 (16.9%)	20 (20.0%)
Forced delivery (vaginal)	55 (10.5%)	27 (11.4%)	13 (9.6%)	14 (14.0%)
1-minute Apgar score	9 [8, 9]	8 [8, 9]	8 [7, 9]	8 [8, 9]
5-minute Apgar score	9 [9, 10]	9 [9, 9]	9 [8, 9]	9 [9, 9]
Night time/weekend referral	256 (48.8%)	118 (50.0%)	70 (51.5%)	48 (48.0%)
Distance of transportation (km)	17.8 ± 17.2	16.5 ± 16.3	16.3 ± 16.3	16.7 ± 16.1
Postnatal age at referral (h)	17.0 ± 18.1	15.5 ± 17.9	16.0 ± 19.1	14.8 ± 16.2
**Primary clinical problem at referral**				
Respiratory problem	198 (37.7%)	83 (35.2%)	46 (33.8%)	37 (37.0%)
Gastrointestinal problem	70 (13.3%)	33 (14.0%)	24 (17.6%)	9 (9.0%)
Preterm birth/low-birth-weight	69 (13.1%)	34 (14.4%)	15 (11.0%)	19 (19.0%)
Cardiac problem	44 (8.4%)	19 (8.1%)	11 (8.1%)	8 (8.0%)
Congenital anomaly	31 (5.9%)	7 (3.0%)	3 (2.2%)	4 (4.0%)
Jaundice	17 (3.2%)	19 (8.1%)	10 (7.4%)	9 (9.0%)
Hypothermia and not being well signs	24 (4.6%)	11 (4.7%)	10 (7.4%)	1 (1.0%)
Fever/infection	21 (4.0%)	10 (4.2%)	4 (2.9%)	6 (6.0%)
Birth asphyxia	17 (3.2%)	9 (3.8%)	7 (5.1%)	2 (2.0%)
Hypoglycaemia	18 (3.4%)	6 (2.5%)	2 (1.5%)	4 (4.0%)
Neurological problem	14 (2.7%)	5 (2.1%)	4 (2.9%)	1 (1.0%)
Other problems	2 (0.4%)	0 (0.0%)	0 (0.0%)	0 (0.0%)
**Risk estimation at referral**				
Estimated as clinically unstable	206 (39.2%)	113 (47.9%)	61 (44.9%)	52 (52.0%)
**Requirement for respiratory support after admission**				
IPPV	91 (17.3%)	59 (25.0%)	33 (24.3%)	26 (26.0%)
IPPV or NIPPV	122 (23.2%)	73 (30.9%)	39 (28.7%)	34 (34.0%)

Values are number (%), mean ± standard deviation or mean [95% confidence interval]. Abbreviations: IPPV, invasive positive pressure ventilation; NIPPV, non-invasive positive-pressure ventilation.

### Estimation of clinically unstable newborns who required IPPV

Between periods 1 and 2, the sensitivity and specificity of the estimation of clinically unstable newborns at referral changed from 0.758 to 0.898 and from 0.684 to 0.661, respectively (Table 2). The false positive rate, false negative rate and positive likelihood ratio were statistically invariant between periods 1 and 2. A true-positive prediction was associated with period 2 (vs. period 1; p = 0.001), caesarean birth, younger gestational age, lower 1- and 5-minute Apgar scores, younger postnatal age, respiratory problem as the primary clinical problem and the interaction between video call and respiratory problem as the primary clinical problem at referral (all p < 0.001) (Table 3). The multivariate model comprised period 2 (p = 0.004), caesarean birth (p = 0.013), younger gestational age (p = 0.001), lower 5-minute Apgar score (p < 0.001), younger postnatal age (p < 0.001) and respiratory problem (p < 0.001).

**Table 2:** Accuracy of video and non-video call–based estimations of respiratory conditions.

	Period 1	Period 2		
		Whole cohort	a Video call (−)	b Video call (+)
	n = 525	n = 236	n = 136	n = 100
**Risk estimation at referral**				
Estimated as clinically unstable	206	113	61	52
**Requirement for respiratory support after admission**				
IPPV	69 (33.5%)	53 (46.9%)	31 (50.8%)	22 (42.3%)
IPPV or NIPPV	86 (41.7%)	61 (54.0%)	35 (57.4%)	26 (50.0%)
None	120 (58.3%)	52 (46.0%)	26 (42.6%)	26 (50.0%)
Estimated as clinically unstable	319	123	75	48
**Requirement for respiratory support after admission**				
IPPV	22 (6.9%)	6 (4.9%)	2 (2.7%)	4 (8.3%)
IPPV or NIPPV	36 (11.3%)	12 (9.8%)	4 (5.3%)	8 (16.7%)
None	283 (88.7%)	111 (90.2%)	71 (94.7%)	40 (83.3%)
**Accuracy of risk-estimation for infants requiring IPPV**				
Sensitivity	0.758 [0.670–0.846]	0.898 [0.821–0.975]	0.939 [0.858–1.021]	0.846 [0.707–0.985]
Specificity	0.684 [0.641–0.728]	0.661 [0.591–0.731]	0.709 [0.621–0.796]	0.595 [0.483–0.706]
False positive rate	0.316 [0.272–0.359]	0.339 [0.269–0.409]	0.291 [0.204–0.379]	0.405 [0.294–0.517]
False negative rate	0.242 [0.154–0.289]	0.102 [0.025–0.155]	0.061 [-0.021–0.115]	0.154 [0.015–0.256]
Positive likelihood ratio	2.402 [2.005–2.878]	2.650 [2.120–3.312]	3.225 [2.357–4.413]	2.087 [1.514–2.877]
**Accuracy of risk-estimation for infants requiring IPPV or NIPPV**				
Sensitivity	0.705 [0.624–0.786]	0.836 [0.751–0.921]	0.897 [0.802–0.993]	0.765 [0.622–0.907]
Specificity	0.702 [0.658–0.747]	0.681 [0.609–0.753]	0.732 [0.644–0.820]	0.606 [0.488–0.724]
False positive rate	0.298 [0.253–0.342]	0.319 [0.247–0.391]	0.268 [0.180–0.356]	0.394 [0.276–0.512]
False negative rate	0.295 [0.214–0.345]	0.164 [0.079–0.230]	0.103 [0.007–0.171]	0.235 [0.093–0.355]
Positive likelihood ratio	2.367 [1.960–2.859]	2.619 [2.048–3.351]	3.348 [2.370–4.730]	1.941 [1.364–2.762]

Values are number (%) or mean [95% confidence interval].

Abbreviations: IPPV, invasive positive pressure ventilation; NIPPV, non-invasive positive-pressure ventilation.

**Table 3:** Independent variables of true-positive predictions of newborns with respiratory dysfunction.

	Predicting requirement for IPPV		Predicting requirement for NIPPV	
	OR [mean, 95% CI]	p-value	OR [mean, 95% CI]	p-value
**Univariate analysis**				
Period 2 (vs. Period 1)	1.914 [1.287–2.847]	0.001	1.779 [1.227–2.581]	0.002
Video call	1.582 [0.942–2.658]	0.083	1.568 [0.962–2.555]	0.071
Caesarean section	2.464 [1.647–3.688]	<0.001	2.710 [1.858–3.954]	<0.001
Gestational age (week)	0.767 [0.703–0.837]	<0.001	0.755 [0.693–0.822]	<0.001
Body weight at birth (g)	1.000 [0.999–1.000]	0.123	1.000 [0.999–1.000]	0.198
5-minute Apgar score	0.566 [0.488–0.656]	<0.001	0.581 [0.503–0.672]	<0.001
Night time/weekend referral	0.963 [0.654–1.419]	0.850	1.026 [0.716–1.470]	0.890
Distance of transportation (km)	1.011 [1.000–1.022]	0.052	1.010 [1.000–1.021]	0.044
Postnatal age at referral (h)	0.946 [0.927–0.965]	<0.001	0.941 [0.924–0.959]	<0.001
**Primary clinical problem at referral**				
Respiratory problem	2.519 [1.700–3.732]	<0.001	2.289 [1.589–3.297]	<0.001
Gastrointestinal problem	0.575 [0.298–1.108]	0.098	0.662 [0.371–1.180]	0.162
Preterm birth/low-birth-weight	0.480 [0.235–0.980]	0.044	0.646 [0.356–1.173]	0.151
Cardiac problem	0.745 [0.346–1.607]	0.453	0.874 [0.444–1.720]	0.697
Congenital anomaly	0.603 [0.210–1.732]	0.347	0.477 [0.167–1.366]	0.168
Jaundice	0.138 [0.019–1.019]	0.052	0.110 [0.015–0.809]	0.030
Hypothermia/not being well signs	1.050 [0.427–2.579]	0.915	0.828 [0.338–2.029]	0.680
Fever/infection	0.168 [0.023–1.242]	0.081	0.133 [0.018–0.986]	0.048
Birth asphyxia	1.986 [0.816–4.833]	0.130	2.290 [1.000–5.247]	0.050
Video call x Respiratory problem	4.370 [2.231–8.558]	<0.001	5.011 [2.542–9.880]	<0.001
**Multivariate analysis**				
Respiratory problem	2.648 [1.693–4.142]	<0.001	2.411 [1.587–3.664]	<0.001
Period 2 (vs. Period 1)	1.973 [1.245–3.126]	0.004	1.840 [1.191–2.842]	0.006
Gestational age (week)	0.799 [0.721–0.886]	<0.001	0.785 [0.711–0.866]	<0.001
5-minute Apgar score	0.631 [0.537–0.740]	<0.001	0.661 [0.564–0.773]	<0.001
Caesarean section	1.808 [1.133–2.884]	0.013	2.099 [1.357–3.246]	0.001
Distance of transportation (km)	1.012 [1.000–1.025]	0.060	1.011 [0.999–1.024]	0.062
Postnatal age at referral (h)	0.967 [0.949–0.986]	0.001	0.961 [0.943–0.979]	<0.001

Abbreviations: IPPV, invasive positive pressure ventilation; NIPPV, non-invasive positive-pressure ventilation; OR, odds ratio; CI, confidence interval.

### Estimation of clinically unstable newborns who required NIPPV

The sensitivity and specificity of predicting clinically unstable newborns at referral changed from 0.705 to 0.836 and from 0.702 to 0.681, respectively (Table 2). The false positive rate, false negative rate and positive likelihood ratio were also statistically invariant between periods 1 and 2. The true-positive prediction was associated with factors such as period 2 (vs. period 1; p = 0.002), caesarean birth, younger gestational age, lower 1- and 5-minute Apgar scores, younger postnatal age at referral, respiratory problem as the primary clinical problem at referral and the interaction between video call and respiratory problem as the primary clinical problem at referral (all p < 0.001; Table 3). The multivariate model included period 2 (p = 0.006), caesarean birth (p = 0.001), younger gestational age (p < 0.001), lower 5-minute Apgar score (p < 0.001), younger postnatal age (p < 0.001) and respiratory problem (p < 0.001).

## Discussion

A range of video call–based communication systems have been proposed to assist with remote diagnosis of foetuses and newborns^20,24,25^. However, few systems were extensively used beyond the initial operation period/region^19^. Several explanations are possible. First, continuous use of these systems involves a substantial cost to maintain the hardware/software^18,23^. Second, referring units generally have only limited opportunities to make video call–based consultations, thus leaving the local staff members unfamiliar with the system. Indeed, during period 2, St. Mary’s Hospital performed 100 video call–based assessments, which account for only 0-4 cases per birth centre. During another project, our team aimed to establish an infection surveillance system within a region damaged after the Great East Japan Earthquake and Tsunami Disaster^26^, and we reported the importance of being able to use locally active communication tools to maintain a high response rate of participating clinics. Although that situation was different from that involving consultations for ill newborns, the development of communication tools for emergencies may benefit from the use of pre-existing tools that involve ordinal operations.

Using free or commercially available video-call applications, we were able to minimise the installation cost of the system to approximately 1,200USD, which was the cost of a desktop computer with a camera, microphone and speaker. Initially, we designed our system to be compatible with diverse communication tools available at each site. However, unexpectedly, all except for two birth centres used FaceTime presumably reflecting the popularity of the iPhone (Apple Inc., Cupertino, CA, USA) in Japan; this accelerated our move to deploy relatively more secure applications. By the end of 2016, the use of video applications with unsecure registration and authentication systems (i.e. Skype, Google Hangouts, Facebook Messenger and Line Phone) was terminated, and the system was operated using FaceTime and Zoom. Because a large volume of personal information can be shared on video calls, continuous effort is required to protect the privacy of newborns and their families. Although dissemination of NCPR contributes to the improvement of resuscitation at birth centres, following the initial treatment, difficult decisions remain regarding the handling of unstable newborns. Objective explanation of newborns’ condition to NICU is also difficult, occasionally leading to insufficient/excessive triage of newborns^27^. To make the video call–based triage more objective, we used a visual assessment tool for respiratory conditions^8^, and the combination of which led to relatively higher sensitivity and lower false-negative rates when predicting newborns with covert respiratory problems. A strategy, that simultaneously achieves the safety of newborns, reduces medical costs and prevents clinically unnecessary mother-newborn separation, might be established with further modifications of our system.

Even during period 2 of our study, video calls were not mandatory for newborns, whose clinical condition was evident from the voice call. This policy might be relevant considering that, at small birth centres, obstetricians/midwives are able to make video calls only after the clinical condition of the newborn is stabilised. However, with additional hands to assist with video calls during resuscitation, neonatologists at a remote centre may provide a real-time feedback to the birth centre regarding the assessment, intervention and other management of the newborn^20^. Future studies need to assess the benefit of using video calls for all newborns regardless of the type and severity of the primary clinical problem.

## Limitations

First, findings from the current historical observational study are susceptible to uncontrolled bias. For example, from period 1 to 2, the number of outborn admissions reduced from 9.7 to 8.4 per month, whereas newborns who required IPPV or NIPPV increased from 23.2% to 30.9%. Given that no major change was introduced to the strategy of respiratory support, and that the occupancy rate of intensive cots remained high throughout the study period, it is likely that the intensive cot was predominantly occupied by relatively more unstable newborns, leaving fewer cots for their less critical peers. Our triage system assessed only respiratory disorders, assuming that non-respiratory conditions may be followed by abnormal respiratory patterns when the newborn’s condition deteriorates. Indeed, approximately 20% of newborns, whose primary symptom at referral was non-respiratory problems, required IPPV or NIPPV. Nevertheless, future studies need to investigate whether inclusion of non-respiratory symptoms within the triage system might improve the quality of assessment. Long-term outcomes were not assessed, which needs to be addressed to confirm the benefits of using video call–based triage. The accuracy could not be reviewed because of the difficulty in recording the video call. For the safety reason, we did not use the feedback from video calls to reduce the number of transportations. Therefore, we were unable to assess whether our strategy or video-call itself helped reduce unnecessary transportation and subsequent separation of the mother and newborn.

## Conclusions

A video call-based consultation system for newborns was successfully developed and operated in a mixed area with urban, rural and remote regions using personal smartphones, which provided obstetricians and midwives at local birth centres with the access to specialists in neonatal medicine. With further improvement of the triage system and video communication tool, our strategy might provide a method of ensuring the safety of ill newborns that involves minimum expense for the installation and maintenance of the hardware, software, network and human resources. Future studies need to assess whether the video consultation system improves the prediction and management of clinically unstable newborns.

## Patients and Methods

**Figure 2:**
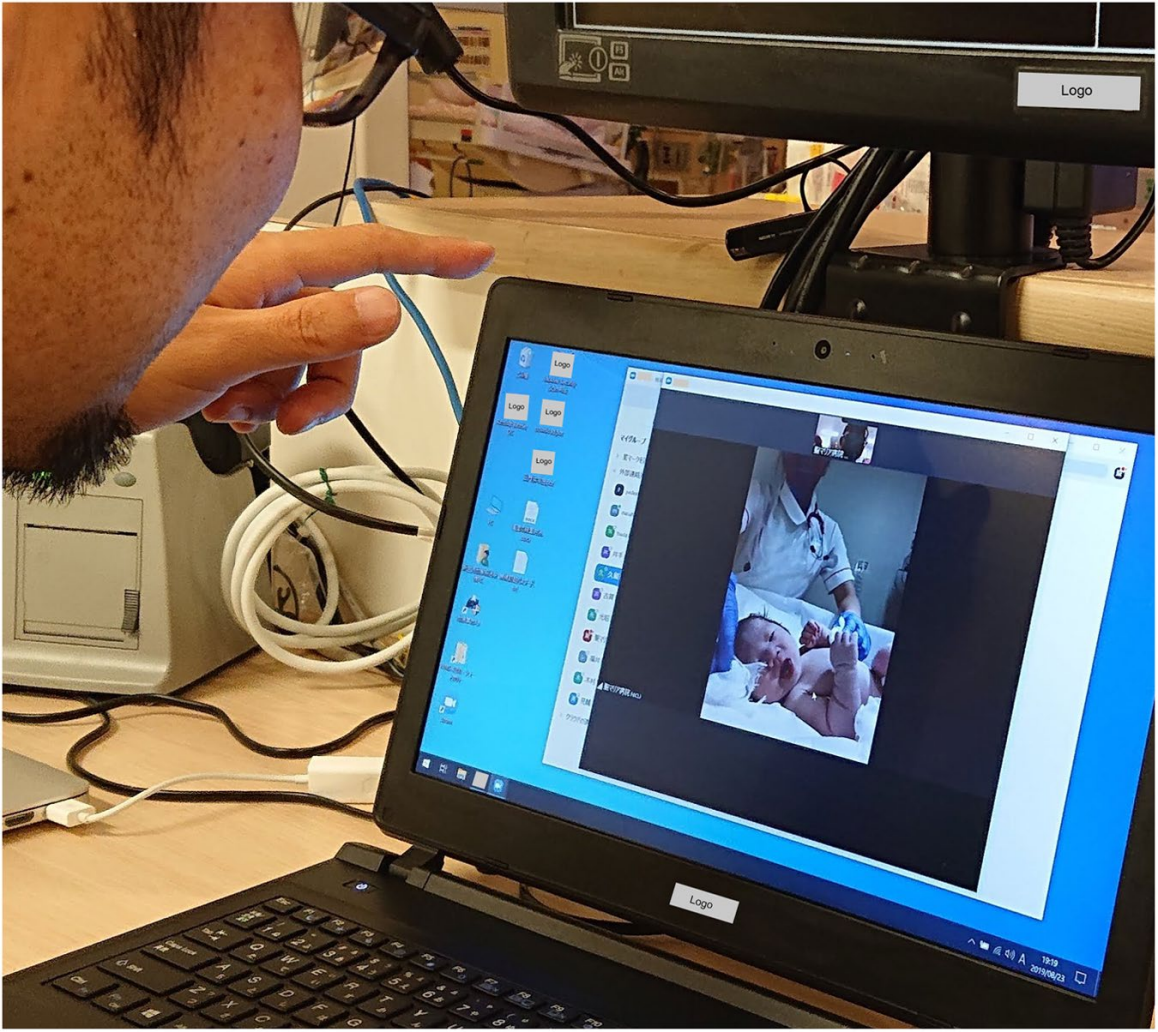
Image depicting the video-based consultation of a newborn infant. A consultant neonatologist of St. Mary’s Hospital assessing the respiratory condition of a newborn infant using the video consultation system (photograph presented with permission of the individuals). The original photograph was edited to mask the name of operation systems and commercial logos.

This retrospective observational study was conducted in compliance with the Declaration of Helsinki under the approval of the Ethics Committee of St. Mary’s Hospital. The Ethics Committee advised that informed consent was unnecessary because only anonymised data obtained for the clinical reason were used in the study. However, informed consent to publish the image in an online open-access publication was obtained from a parent of a newborn infant, who appears in Fig. 2.

Between April 2011 and December 2017, 3,097 newborns were admitted to a tertiary NICU at St. Mary’s Hospital (Kurume, Fukuoka, Japan). St. Mary’s Hospital includes one of two tertiary NICUs in the area, which comprises a population of 700,000 in the southern half of Fukuoka Prefecture and boundary parts of Saga, Nagasaki, Kumamoto and Ohita Prefectures. Transportation teams are formed in response to the consultation from birth centres according to the estimated severity of newborns (i.e. a driver/porter and a nurse for clinically stable newborns and a driver/porter, nurse and a neonatologist for clinically unstable newborns).

Of 911 newborns transferred to St. Mary’s Hospital during the study period, newborns who were referred 72 h after birth (n = 136), those for whom consultation was performed before birth (n = 9) and those who were unexpectedly delivered at home or in the ambulance (n = 5) were excluded from the analysis, leaving 761 newborns available as the study population (Fig. 1). The neonatologists/paediatricians at St. Mary’s Hospital were asked to identify clinically unstable newborns, who are likely to benefit from respiratory support during transportation, based on the information obtained from the consultation. The study period was divided into two. During period 1 (April 2011 to August 2015), the voice call was used for consultations. During period 2 (September 2015 to December 2017), after the initial consultation using the voice call, the video call was performed except in cases when the newborn’s condition appeared to be clinically stable after the voice call or when the newborn’s condition appeared to be clinically unstable and required urgent transportation (Fig. 2). During the video call, obstetricians at the birth centres were asked to provide two views of the newborn in the supine position for at least 20 seconds. The first view involved the camera looking down at the newborn from either side to determine the presence of nasal flaring and retractions on the trunk. The second view focused on the newborn’s chest/abdominal wall motion. To standardise the video-based triage, the neonatologists/paediatricians at St. Mary’s Hospital attended at least one training session to learn the algorithm of the visual respiratory function assessment scale, which was originally developed to identify newborns with low dynamic respiratory compliance (<0.6 mL/cmH_2_O/kg)^8^. Newborns with a respiratory rate >50/min and at least one of the following were considered to be clinically unstable: nasal flaring, see-saw respiration and suprasternal/intercostal retraction. We did not consider non-respiratory symptoms, because respiratory dysfunction is a major reason for neonatal referral and non-respiratory conditions may ultimately show abnormal respiratory patterns when the systemic condition deteriorates.

In June 2015, an invitation letter with instructions explaining how to perform video calls with the NICU at St. Mary’s Hospital was sent to birth centres from where at least one newborn had been referred over the past 5 years. The participating units were allowed to use various devices (e.g. internet-connected personal computers, electronic pads and smartphones) and applications (i.e. Google Hangouts, FaceTime, Skype, Line Phone, Facebook Messenger and Zoom). Step-by-step instructions for each device and application were also provided. For approximately 20% of the heads of birth centres whose mobile phones were incompatible with the aforementioned applications, we advised one of the staff members at the birth centre who owns a personal smartphone to make the video call.

### Clinical information

Clinical information from the electronic patient records including gestational age, birth weight, sex, delivery mode, Apgar scores, time of consultation, primary clinical problem, distance of transportation, postnatal age, estimation of clinical condition (stable/unstable) and requirement of respiratory support during or <6 h after transportation was collected. The time of initial consultations were categorised into two groups: weekday daytime and others (i.e. night time, 18:00-08:00; weekend, Saturday and Sunday; national holidays). The primary clinical problem at referral was grouped as follows: respiratory problems (including dyspnoea, respiratory distress, apnoea and insufficient oxygenation); gastrointestinal problems (including vomiting, poor feeding and unfavourable passage of milk); preterm birth and/or low birth weight; cardiac problems (including cardiac dysfunction, heart murmur, arrhythmia and suspicion of congenital heart diseases); congenital anomalies; jaundice; hypothermia and signs of being unwell; fever and/or infection; birth asphyxia; hypoglycaemia; neurological problems such as seizures; and other disorders.

### Data analysis

Background clinical variables were compared between periods 1 and 2 and between the subcohorts of period 2 (period 2a: did not perform a video call; period 2b: performed a video call) using the chi-square test, Fisher’s exact test or Student’s t-test with Bonferroni correction. Sensitivity, specificity, true-positive rate, true-negative rate, false-positive rate and false-negative rate of the risk estimation at the time of initial consultations compared to the subsequent requirement for invasive positive-pressure ventilation (IPPV) and non-invasive positive-pressure ventilation (NIPPV) during or <6 h after transportation were obtained for each cohort/subcohort. To assess whether the introduction of the video call system is associated with altered true-positive triage of clinically unstable newborns, univariate and multivariate logistic regression analyses were performed. Findings from the univariate logistic regression analysis were not corrected for multiple comparisons, however, p-values between 0.01 and 0.05 were regarded as chance level.

## Data Availability

The datasets generated during and/or analysed during the current study (excluding those potentially leading to the identification of personal information) are available from the corresponding author upon reasonable request.
